# DeepTSS: multi-branch convolutional neural network for transcription start site identification from CAGE data

**DOI:** 10.1186/s12859-022-04945-y

**Published:** 2022-12-12

**Authors:** Dimitris Grigoriadis, Nikos Perdikopanis, Georgios K. Georgakilas, Artemis G. Hatzigeorgiou

**Affiliations:** 1 Hellenic Pasteur Institute, 11521 Athens, Greece; 2 Department of Computer Science and Biomedical Informatics, University of Thessaly, 35131 Lamia, Greece; 3 Department of Informatics and Telecommunications, National and Kapodistrian University of Athens, 15784 Athens, Greece; 4 Department of Electrical and Computer Engineering, University of Thessaly, 38221 Volos, Greece; 5 ommAI Technologies, Tallinn, Estonia

**Keywords:** TSS, CAGE, Bioinformatics, Promoter, Transcription, Machine Learning, Deep Learning, GSP

## Abstract

**Background:**

The widespread usage of Cap Analysis of Gene Expression (CAGE) has led to numerous breakthroughs in understanding the transcription mechanisms. Recent evidence in the literature, however, suggests that CAGE suffers from transcriptional and technical noise. Regardless of the sample quality, there is a significant number of CAGE peaks that are not associated with transcription initiation events. This type of signal is typically attributed to technical noise and more frequently to random five-prime capping or transcription bioproducts. Thus, the need for computational methods emerges, that can accurately increase the signal-to-noise ratio in CAGE data, resulting in error-free transcription start site (TSS) annotation and quantification of regulatory region usage. In this study, we present DeepTSS, a novel computational method for processing CAGE samples, that combines genomic signal processing (GSP), structural DNA features, evolutionary conservation evidence and raw DNA sequence with Deep Learning (DL) to provide single-nucleotide TSS predictions with unprecedented levels of performance.

**Results:**

To evaluate DeepTSS, we utilized experimental data, protein-coding gene annotations and computationally-derived genome segmentations by chromatin states. DeepTSS was found to outperform existing algorithms on all benchmarks, achieving 98% precision and 96% sensitivity (accuracy 95.4%) on the protein-coding gene strategy, with 96.66% of its positive predictions overlapping active chromatin, 98.27% and 92.04% co-localized with at least one transcription factor and H3K4me3 peak.

**Conclusions:**

CAGE is a key protocol in deciphering the language of transcription, however, as every experimental protocol, it suffers from biological and technical noise that can severely affect downstream analyses. DeepTSS is a novel DL-based method for effectively removing noisy CAGE signal. In contrast to existing software, DeepTSS does not require feature selection since the embedded convolutional layers can readily identify patterns and only utilize the important ones for the classification task.

This study highlights the key role that DL can play in Molecular Biology, by removing the inherent flaws of experimental protocols, that form the backbone of contemporary research. Here, we show how DeepTSS can unleash the full potential of an already popular and mature method such as CAGE, and push the boundaries of coding and non-coding gene expression regulator research even further.

**Supplementary Information:**

The online version contains supplementary material available at 10.1186/s12859-022-04945-y.

## Background

With the advent of Next Generation Sequencing (NGS) in the early twenty-first century, the scientific fields related to transcriptional dynamics have experienced significant breakthroughs at an unprecedented rate. In 2003, Cap Analysis of Gene Expression (CAGE) was introduced as an experimental method that is able to capture and quantify the 5’ end of capped RNA, making it an ideal tool for transcriptomic studies [1]. CAGE has gradually been improved into a state-of-the-art approach for genome-wide promoter and enhancer characterization. The FANTOM consortium [2] has been pioneering the CAGE refinement effort by sequencing thousands of CAGE samples.

**Fig. 1:**
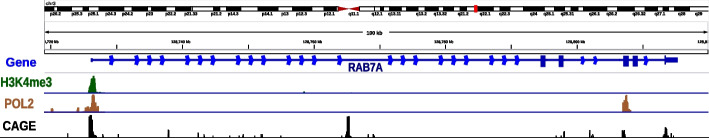
RAB7A gene locus shown as an example of the noisy signal embedded in CAGE samples. Besides the CAGE peak that is located on the annotated TSS of RAB7A, there are numerous tag clusters localized upstream and especially within the intronic as well as the exonic regions. Ideally, every TSS identification algorithm that utilizes CAGE, should be able to accurately remove the noise and only keep CAGE peaks that correspond to transcription initiation events

This volume of information has been systematically used for the past two decades to characterize the regulatory genomic regions of model organisms and to advance our knowledge about the gene regulatory networks implicated in numerous diseases. However, recent studies have revealed that CAGE samples inherently suffer from significant levels of noise [3–5]. Specifically, the detected noise mostly corresponds to byproducts of the splicing and transcription machineries. Such signal is entirely unrelated to transcription initiation and exists even in high quality samples, suggesting the need for preprocessing of CAGE data to increase the specificity of this protocol for transcription start site (TSS) identification (Fig. 1).

The need to remove the aforementioned noise and maintain crucial TSS-related signal, has led to the development of computational methods that, given a CAGE sample already aligned on the genome, they provide a list of signal enriched regions. For some of these algorithms, the selection of the provided CAGE peaks is based on statistical methods while for others on Machine Learning (ML). CAGER [3] and RECLU [4] belong in the first category of algorithms that remove noise by statistically analyzing its reproducibility across replicated experiments. PARACLU [1] operates in a more simplistic way by aggregating reads into peaks using certain distance or expression criteria. The TOMETOOLS suite [2] includes a classifier that is able to filter out non-TSS associated CAGE signal, based on Gaussian mixture models and a random decision tree ensemble. iTiSS [6] performs a joint analysis of several complementary datasets for distinguishing between true and false positive TSSs. ADAPT-CAGE [7] is the latest addition to CAGE processing algorithms, and combines structural and promoter-associated motif features with ML to distinguish between TSS-related signal and transcriptional noise. ADAPT-CAGE uses a stacked modular ML architecture. Each module operates on a separate feature category and forwards its output to the next layer, formulating an agent assembly strategy.

Signal processing methods have long been used in Biology for providing answers related to a multitude of intriguing questions such as exon and sequence structure prediction [8, 9], protein-coding DNA sequence identification [10] and the localization of repeat elements [11]. When applied to biological problems, these methods are referred to as genomic signal processing (GSP), and they include techniques for transforming the NGS signal and the underlying DNA sequence to numerical vectors in order to be treated as time-series data [12].

Typically, in computational pipelines, GSP is initially applied to extract a set of features that are subsequently used as input to ML algorithms. Even though there are a multitude of genomic studies in the literature that combine GSP with ML [13–15], there is no evidence of how GSP-derived features perform when used as input for Deep Learning (DL) for studying transcription. There is early work that dates back to 1998 [16] that paved the way for DL as we know it today. DL is a subfield of ML that refers to state-of-the-art Neural Network (NN) algorithms that typically include convolutional layers (CNNs) prior to the densely connected part of the architecture [16]. The stacks of convolutional layers can progressively learn primitive to more abstract patterns and forward their decision to a multilayer perceptron type of neuron cluster also referred to as dense layers. Since 2012, and due to the technological advancements of graphics processing units, the training of NNs with dozens of layers and millions of parameters became computationally feasible [17]. This breakthrough has revolutionized numerous scientific fields including Biology, Chemistry, Physics, Pharmacology, Engineering, Economics as well as the commercial and industrial sectors [18–22]. Specifically in Biology, DL has been applied to a wide spectrum of fields such as transcriptomics [23], genomics [24], and epigenetics [25] among others, achieving unprecedented levels of performance.

**Fig. 2:**
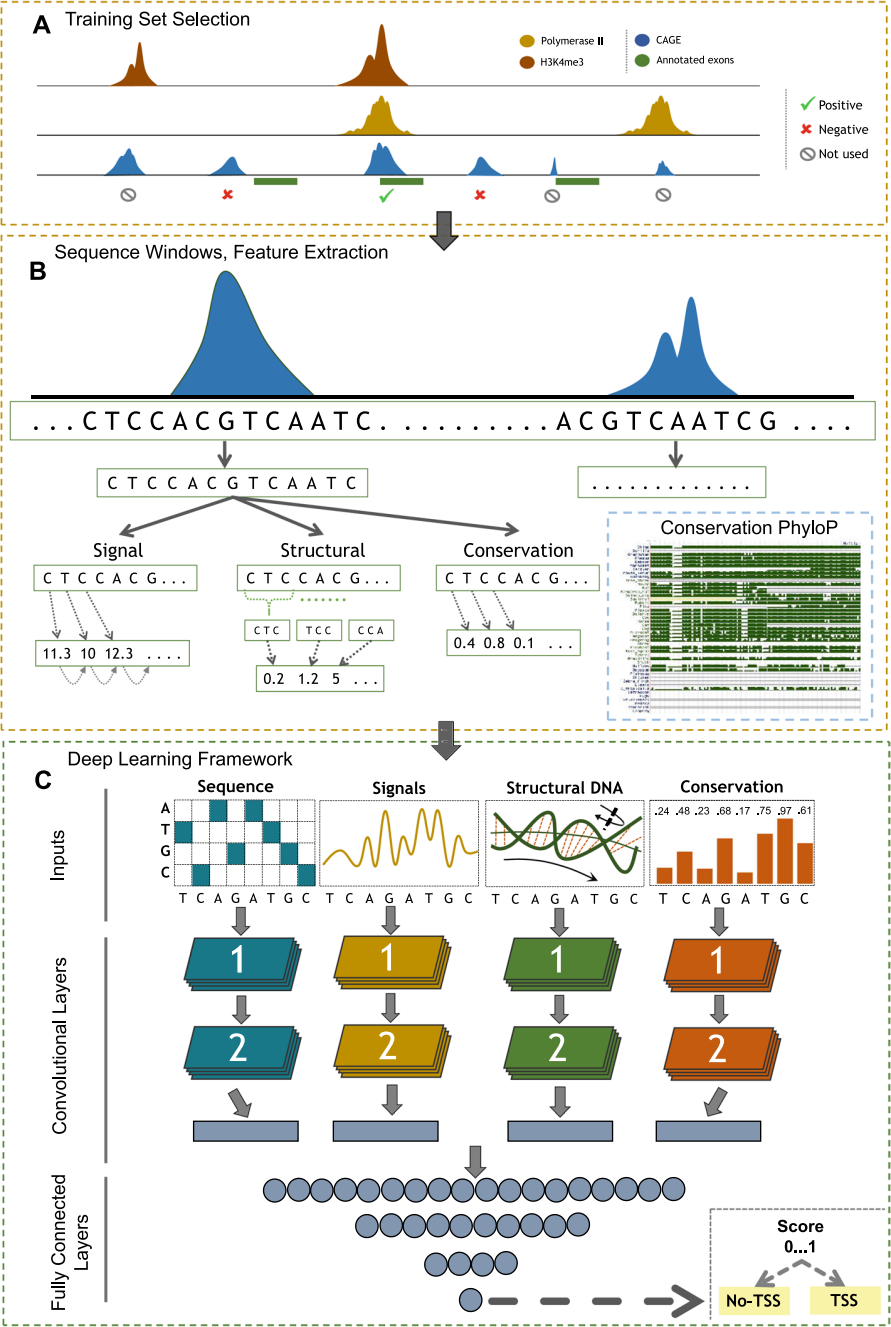
Overview of training set selection, feature extraction and DeepTSS DL architecture. **a** Synopsis of the process for labeling H9 CAGE peaks as positive or negative samples. Peaks exhibiting an overlap with annotated protein-coding gene TSSs as well as both H3K4me3 and Polymerase II enriched loci were labeled as positives while those that overlapped with either of the two marks but not with annotated TSSs were removed from any subsequent analysis. CAGE peaks that did not overlap with any of the two marks and annotated TSSs were marked as negatives. **b** For each peak representative (position with highest amount of overlapping 5’ end of tags) we extracted the centered underlying sequence (600 bp) and proceeded to extract four distinct feature categories. The one-hot encoded version of the sequence, the GSP-inspired and structural DNA sequence-based features as well as the per nucleotide evolutionary conservation evidence. **c** DL architecture of DeepTSS, specifically designed for exploiting each individual feature type. The architecture consists of 4 distinct convolutional branches for processing the different feature types. The first branch operates on the one-hot encoded version of the input DNA sequence, the second and third on GSP and structural DNA features respectively, and the fourth on the evolutionary conservation evidence. All branches are designed with 2 consecutive convolutional layers and their output is concatenated prior to the application of the fully connected part of the network. The final output is based on a sigmoid activation function

In this study, we introduce DeepTSS, an extension of our previous method named DiS-TSS [26] for distinguishing between TSS-associated CAGE signal and biological or technical noise. DeepTSS is a computational framework for accurate and single-nucleotide resolution TSS identification that combines GSP, sequence and evolutionary conservation features, CAGE data and DL (Fig. 2c). After basic pre-processing, aligned tags overlapping with CAGE peaks are transformed to signal vectors in the time domain and GSP-inspired features are calculated. Additionally, the DNA sequence corresponding to CAGE peaks is one-hot encoded and structural features are extracted along with the evolutionary conservation score as calculated by phyloP [27]. Each feature type is provided as input to separate convolutional layers, which is a branching scheme that has already been successfully utilized in the context of precursor microRNA prediction [18]. The output of these layers is concatenated and forwarded to the densely connected part of the architecture. Using a multifaceted benchmarking strategy based on annotated genomic loci and experimental data, DeepTSS was found to outperform previously published algorithms in distinguishing real transcription initiation events from biological and protocol-induced noise. DeepTSS is a Python framework specifically designed for ease-of-use that is freely accessible at https://github.com/DianaLaboratory/DeepTSS.

## Results

Regardless of their common objective, the evaluation process of algorithms with diverse feature extraction processes and mathematical modeling is far from trivial. Our strategy for comparing DeepTSS, ADAPT-CAGE, TOMETOOLS, CAGER, RECLU, PARACLU and iTiSS was specifically designed to calculate an unbiased estimate of each algorithm’s performance. The first benchmark is based only on protein-coding gene annotations that were used to divide the genome into promoter (positive) and non-promoter (negative) regions. The second benchmark consists of purely experimental data including H3K4me3 and transcription factor ChIP-Seq enriched regions as well as a segmentation of the human genome into chromatin states as calculated by ChromHMM [28], downloaded from the Roadmap Epigenomics Project.

DeepTSS was trained on CAGE samples from H9 cells and all benchmarks were based on K562 datasets to query its ability to generalize on unseen data.

### Evaluation based on protein-coding gene annotation

The evaluation of TSS predictors, presented in this section, was based on a benchmark created by annotated protein-coding genes. For each TSS we labeled the proximal region (± 500b) as the positive zone, and the flanking region (± 50.000b) as the negative zone (Additional file 3: Fig. S2). Positive predictions overlapping the positive zones were deemed true positives (TP) and the rest were considered false positives (FP). Any prediction falling within a negative zone and exhibiting an overlap with H3K4me3 ChIP-Seq peaks was flagged as a TP instead of FP.

Positive zone is defined as a ± 500b window centered on the annotated TSS as it has been reported to be occupied by TSSs across different tissues (e.g. alternative TSSs) [29] or under different conditions [30].

To also observe the performance based on a different point of view, instead of evaluating each CAGE peak individually, we considered that a positive prediction is a gene exhibiting at least one overlapping CAGE peak (algorithms’ output) with its TSS vicinity (± 500 bp).

**Fig. 3:**
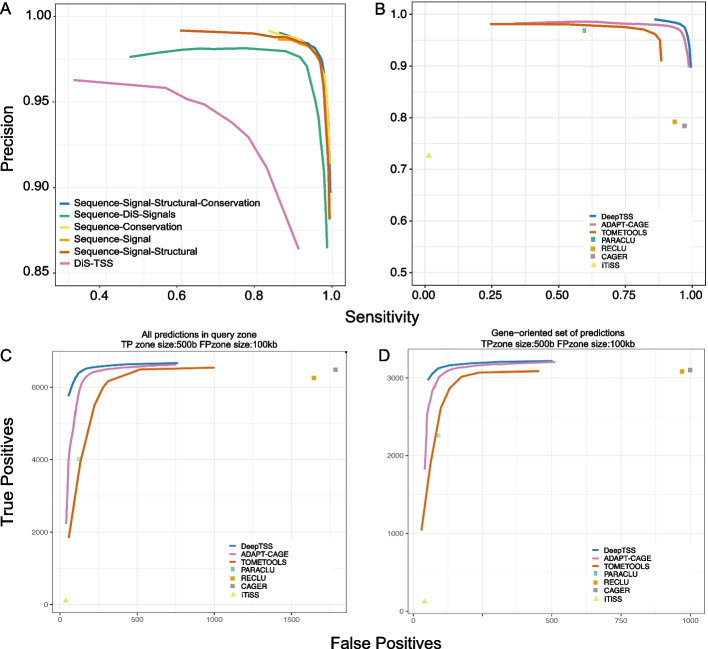
Annotation-based evaluation in K562 cells, based on known protein-coding TSSs. For all algorithms, we applied multiple score cutoffs to calculate performance metrics in a wide range of prediction confidence and generate Precision-Sensitivity as well as TP-FP curves. **a** Comparison of distinct DeepTSS models trained with different combinations of input feature types and our initial GSP-inspired algorithm, DiS-TSS [26], that used SVM to model the data. The green curve corresponds to a DL model trained on the GSP features used in DiS-TSS and the one-hot encoded version of the raw DNA sequence. **b** DeepTSS, ADAPT-CAGE and TOMETOOLS performance as measured with precision and sensitivity. Trade-off between TPs and FPs in the CAGE peak- (**c**) and gene-oriented (**d**) evaluation

In the case of DeepTSS and ADAPT-CAGE, we applied several score cutoffs to explore their performance in the full score range (Fig. 3). PARACLU, RECLU, CAGER and iTiSS do not provide a score for every CAGE peak. Instead, their output is the equivalent of DeepTSS and ADAPT-CAGE predictions after applying a score cutoff. For this reason, precision-recall curves were not calculated for PARACLU, RECLU, CAGER and iTiSS, which are denoted as points in the graphs. A comparison of all algorithms on this benchmark is also presented in Table 1. PARACLU, RECLU, CAGER and ADAPT-CAGE were used with default settings, and DeepTSS with a score cutoff of 0.9.

**Table 1:** Evaluation results in K562 cells, based on annotated protein-coding gene TSSs. From left to right, the number of total positive predictions of each algorithm is shown, the number of TPs and FPs in both CAGE- and gene-oriented benchmarks as well as the performance in terms of precision and sensitivity on default parameters

Algorithm	Total positive predictions	All predictions in query zone		Gene − oriented set of predictions		Protein-coding TSS annotation	
		TP	FP	TP	FP	Precision	Sensitivity
DeepTSS	31,443	6398	123	3122	91	0.98	0.96
ADAPT-CAGE	31,177	6294	172	3091	125	0.97	0.94
CAGER	14,465	6489	1771	3102	1003	0.97	0.78
PARACLU	9453	4016	129	2258	95	0.97	0.60
RECLU	11,558	6257	1649	3082	970	0.93	0.79
TOMETOOLS	30,689	5765	228	3016	174	0.96	0.86
iTiSS	1734	98	37	118	40	0.72	0.01

In Fig. 3a we observe the performance, in terms of precision and sensitivity, of various DeepTSS models that were trained on multiple combinations of the four input features types (Fig. 2c), denoted as Sequence-Signal, Sequence-Conservation, Sequence-Signal-Structural and Sequence-Signal-Structural-Conservation. In addition, we have added our initial GSP-inspired algorithm, DiS-TSS [26], to showcase the difference in performance after adding more features and by using DL instead of SVM for modelling the data. We also added a DL model trained on the GSP signals that were used in DiS-TSS study and the one-hot encoded version of the raw DNA sequence, denoted as Sequence-DiS-TSS. The best performing DeepTSS model was the one trained with all four feature types and was considered our final model for all subsequent analyses and comparisons. The comparison between DeepTSS, ADAPT-CAGE and TOMETOOLS is shown in Fig. 3b (Additional file 1: Table S1). The number of TPs and FPs after applying multiple thresholds can be seen in Fig. 3c (Additional file 1: Table S1). The performance on the gene-oriented approach is presented in Fig. 3d (Additional file 1: Table S2).

**Fig. 4:**
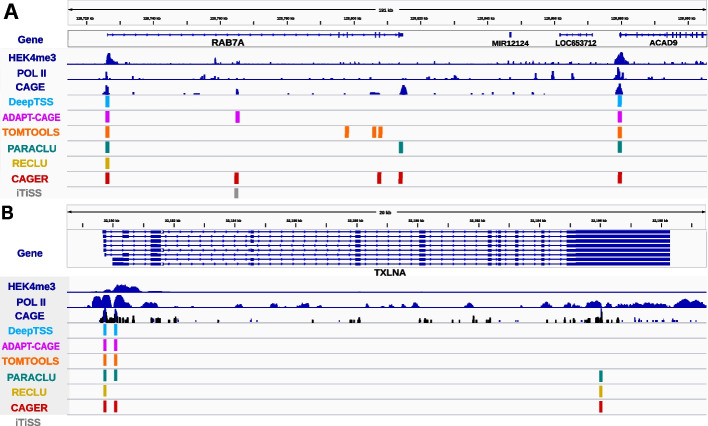
Example of each algorithm’s capacity for accurately removing noise from CAGE data. We have added tracks of experimental evidence (Polymerase II and H3K4me3) to highlight the CAGE signal that corresponds to the transcription initiation site of **a** RAB7A and **b** TXLNA. The prediction of each algorithm is shown on separate tracks

In Fig. 4, the RAB7A and TXLNA locus is shown as an example that highlights the capacity of each algorithm to accurately remove noise from CAGE data.

### Evaluation based on ChromHMM-derived genome annotation and experimental data

**Fig. 5:**
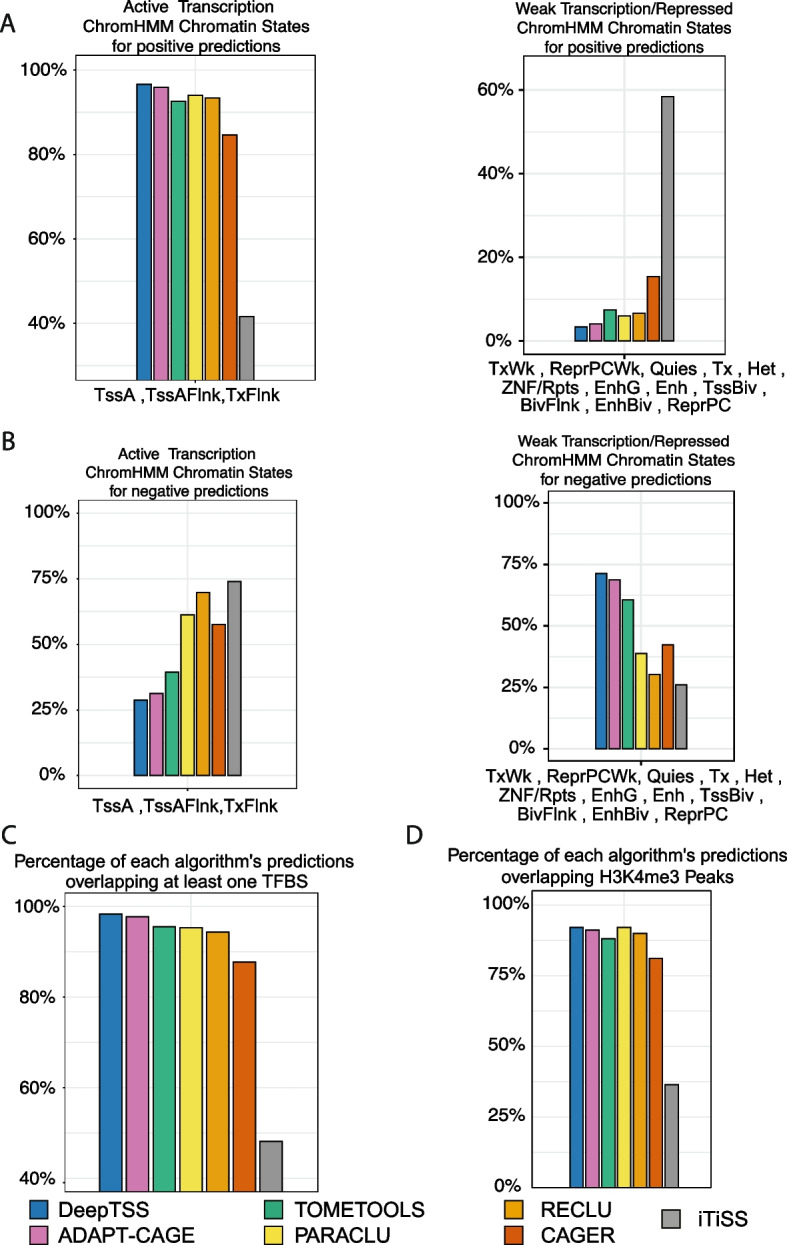
Evaluation of algorithms based on H3K4me3 and TF ChIP-Seq peaks as well as ChromHMM-derived chromatin states from the analysis of six histone modifications in K562 cells. Percentage of each algorithm’s positive (**a**) and negative (**b**) predictions overlapping chromatin states associated with genomic regions exhibiting active (left panel) and weak/repressed (right panel) transcription. Percentage of the algorithms’ positive predictions with at least one TFBS (**c**) and H3K4me3 peak (**d**) derived from ChIP-Seq

In this section we present the comparison results based on two evaluation benchmarks on K562 cells (Additional file 1: Tables S3 and S6A–D). The first one is based on ChromHMM, a well-established ML algorithm for genome segmentation on different chromatin states from the analysis of six histone modifications (Fig. 5a, b, Additional file 2: Fig. S1A, B). The second is based on purely experimental data related to H3K4me3 and TFBS occupancy (Fig. 5c, d, Additional file 2: Fig. S1C, D). We applied a 0.9 score cutoff on DeepTSS and default settings (Additional file 1: Table S4) on ADAPT-CAGE, TOMETOOLS, PARACLU, RECLU, CAGE and iTiSS for isolating their positive predictions. A summary of the comparison results of this evaluation process is shown in Table 2.

**Table 2:** Summarized evaluation results in K562 cells, based on chromatin states, experimental data and annotated protein-coding TSSs. From left to right, each column shows the number of total positive predictions of each algorithm, the percentage overlapping with chromatin states associated with active and weak/repressed transcription, and at least one TF and H3K4me3 peak

Algorithm	Total positive predictions	ChromHMM active transcription	ChromHMM weak transcription	TF ChIP-Seq peaks	H3K4me3 ChIP-Seq peaks
DeepTSS	31,443	96.66%	3.33%	98.27%	92.04%
		(30,376)	(1047)	(30,898)	(28,939)
ADAPT-CAGE	31,177	95.90%	4.09%	97.72%	91.12%
		(29,885)	(1276)	(30,466)	(28,409)
CAGER	14,465	84.62%	15.37%	87.67%	81.11%
		(12,195)	(2215)	(12,681)	(11,732)
PARACLU	9453	94.01%	5.98%	95.26%	92.06%
		(8868)	(565)	(9,005)	(8702)
RECLU	11,558	93.35%	6.64%	94.32%	89.96%
		(10,773)	(767)	(10,902)	(10,397)
TOMETOOLS	30,689	92.59%	7.40%	95.46%	88.12%
		(28,395)	(2272)	(29,296)	(27,044)
iTiSS	1734	41.58%	58.41%	48.12%	41.94%
		(721)	(1013)	(848)	(739)

In the first benchmark we calculated the percentage of each algorithm's positive predictions with active transcription (group 1) and repressed or weak transcription chromatin states (group 2) as annotated by ChromHMM (Fig. 5a). 96.66% (30,376) of DeepTSS’s positive predictions were found to overlap group 1 and 3.33% (1047) group 2. ADAPT-CAGE performance was 95.90% (29,885) and 4.09% (1276) for group 1 and 2 respectively, TOMETOOLS 92.59% (28,395) and 7.40% (2272), PARACLU 94.01% (8868) and 5.98% (565), RECLU 93.35% (10,773) and 6.64% (767), CAGER 84.62% (12,195) and 15.37% (2215) and iTiSS 41.58% (721) and 58.41% (1013).

In the case of negative predictions (Fig. 5b), DeepTSS exhibited an overlap of 28.74% (4576) with group 1 and 71.25% (11,343) with group 2, 31.31% (5067) and 68.68% (11,114) for ADAPT-CAGE, 39.42% (6609) and 60.57% (10,155) for TOMETOOLS, 61.25% (18,522) and 38.75% (11,718) for PARACLU, 69.72% (27,147) and 30.27% (11,790) for RECLU, 57.63% (13,643) and 42.36% (10,029) for CAGER and 73.94% (34,405) and 26.05% (12,121) for iTiSS.

For the second evaluation approach we wanted to explore the occupancy of TF and H3K4me3 ChIP-Seq peaks in the vicinity of positive predictions (Fig. 5c, d for TF and H3K4me3 respectively). For DeepTSS, 98.27% (30,898) of positive predictions overlapped with at least one TFBS and 92.04% (28,939) with H3K4me3, ADAPT-CAGE 97.72% (30,466) and 91.12% (28,409), TOMETOOLS 95.46% (29,296) and 88.12% (27,044), PARACLU 95.26% (9005) and 92.06% (8702), RECLU 94.32% (10,902) and 89.96% (10,397), CAGER 87.67% (12,681) and 81.11% (11,732) and iTiSS 48.12% (848) and 41.94% (739).

## Discussion

In multicellular organisms, the diversity of gene expression is a key driver of the functional variability that is observed across distinct cell types. The exploration of the mechanisms responsible for transcription and the identification of the underlying patterns of gene expression regulation, will always be based on highly optimized experimental techniques that provide trustworthy data with minimized levels of noise.

During the past two decades, numerous experimental protocols were developed specifically to facilitate studies related to transcription dynamics, including CAGE-, RNA-, GRO- and ChIP-Seq. Each protocol focuses on different aspects of the transcription process and inherently possesses different types and levels of noise and biases. Regardless, eliminating these biases and minimizing the noize is a procedure that often requires the development of computational methods that utilize statistical models or even ML.

CAGE is a protocol that offers a clear advantage when studying the dynamics related to transcription initiation, alternative promoter usage and the identification of enhancer RNAs. CAGE was initially introduced in 2003 [31], and since then it has been continuously refined by the FANTOM consortium in a process that resulted in thousands of CAGE samples from a wide range of cell types and tissues in human and mouse [2]. The success of CAGE is undeniable, and reflects on the number of high impact studies available in the literature [32–36] that utilized this protocol and the wealth of information available in the FANTOM repository.

The increased popularity of this protocol inevitably resulted in the identification of its drawbacks. The major flaw in this method is associated with the high levels of biological and technical noise that has been systematically identified by recent studies [1, 3, 4, 7]. It becomes apparent that the community needs computational methodologies that filter out the noise without risking the ability of CAGE to capture transcription initiation events.

Several in silico methodologies have already been introduced in the literature that attempt to deal with the aforementioned limitations of CAGE. PARACLU [1], RECLU [4] and CAGER [3] use statistical modeling to identify CAGE peaks that can be reproduced across replicate samples of the same experiment. iTiSS [6] performs a joint analysis of several complementary datasets to remove false positive TSSs. ADAPT-CAGE [7] and TOMETOOLS [2] are the only algorithms that are entirely based on engineering features related to transcriptionally engaged regulatory elements and ML. ADAPT-CAGE has achieved breakthrough performances in removing CAGE signal that does not correspond to transcription initiation events. However, its major limitation is the process of feature engineering since it requires people with domain expertise and can often lead to the unintentional introduction of biases.

In this study we introduce DeepTSS, a novel computational methodology that combines for the first time, GSP, structural DNA features calculated directly from the sequence, the encoded version of raw DNA sequence and evolutionary conservation evidence, with DL. The comparison between DeepTSS and existing algorithms was a highly sensitive process and required meticulous planning, since each in silico approach is fundamentally different from the others both algorithmically and in terms of the underlying mathematical framework. Even though each comparison benchmark in our threefold strategy has its own advantages and disadvantages, they perfectly complement each other, and most importantly, they provide a view of the algorithms’ performance under distinct testing environments, unveiling important clues on their applicability and functional limitations.

## Conclusions

CAGE is a key protocol in deciphering the language of transcription dynamics, and its contribution in advancing the field of gene regulatory networks has been instrumental. Despite its popularity, CAGE is not absent from the list of experimental methods that suffer from biological and technical noise that if not removed, can significantly diminish the robustness of downstream analyses.

DeepTSS is a novel DL-based computational framework for removing noise from CAGE data and maximizing the probability that the remaining CAGE signal corresponds to transcription initiation events. DeepTSS operates on a seemingly unrelated spectrum of features that are used as input to a DL architecture that was specifically designed to exploit each individual feature type. In contrast to existing implementations, DeepTSS does not require any kind of prior feature engineering process since it relies on convolutional layers directly embedded in the DL architecture that can readily identify patterns and only utilize the important ones for the classification task. DeepTSS was found to outperform existing state-of-the-art implementations when evaluated on a meticulously designed strategy that included experimental data and high quality genome annotations.

This study highlights the importance of ML, and specifically DL, in providing solutions to removing inherent flaws in experimental methods that are the bread-and-butter of contemporary Molecular Biology research. Reliable algorithms, such as DeepTSS, can unleash the full potential of already popular protocols such as CAGE, and play a fundamental role towards unveiling key gene expression regulators as well as pushing the boundaries of non-coding RNAs implication in regulatory networks even further.

## Methods

### Annotation and experimental data

Pre-aligned CAGE datasets in bam format (GRCh38 assembly), from H9 and K562 cells (CNhs11917 and CNhs12334 sample codes) and the corresponding collapsed CAGE tags (5’ end) contained in ctss files were downloaded from FANTOM repository [2]. DeepTSS and ADAPT-CAGE can either be applied on bam files or bed files with pre-calculated CAGE peak representatives and iTiSS can only be utilized on bam files. In contrast, PARACLU, RECLU and CAGER can only be applied on the ctss files.

ChIP-Seq datasets for Polymerase II and H3K4me3 with ENCFF281VBW, ENCFF773FKD, ENCFF757WPX and ENCFF261REY sample codes were downloaded from the ENCODE repository [37] in bed narrowpeak format and the UCSC liftover software was used to migrate them to GRCh38 assembly coordinates.

Genomic locations of transcription factor binding sites (TFBS) from 161 TFs in 91 cell types were derived from the ENCODE 'Txn Factor' track in UCSC. The protein-coding gene annotation was downloaded from Ensembl v98 [38], and the 100 way phyloP per-nucleotide evolutionary conservation score from UCSC.

### Overview of DeepTSS

Within the DeepTSS framework, users can initially provide pre-aligned CAGE tags in bam format and tags with a lower than a user-defined (default = 10) mapping quality are removed. The remaining tags are grouped into peaks based on distance parameter (default = 50 bp). Subsequently, the expression level (normalized in tags per million—tpm) of each peak is calculated and those below a user-defined cutoff (default = 1) are removed. Within every peak, the position with the highest number of overlapping 5’ tag ends is identified and selected as the peak representative. Alternatively, users can provide their own bed formatted CAGE peak representatives directly as input to DeepTSS and void all the previous steps. DeepTSS will then proceed to extract all the required features from the region surrounding the representatives and apply the DL model to score them.

### Network architecture

DeepTSS utilizes the one-hot encoded DNA sequence surrounding the CAGE peak representatives, GSP and structural features extracted from the sequence, and evolutionary conservation as calculated by phyloP. GSP and structural features are standardized separately for each branch while the conservation score is not processed (zeros are assigned to regions with no score).

For each input category a separate convolutional branch is created, composed of 2 layers (Fig. 2c). The computations from all branches are concatenated and directed to the final fully connected layers of the architecture. All branches operate on a 600 bp window size. We tested a multitude of models with different filter numbers, kernel sizes and nodes in the dense layers and we picked the combination that achieved the best performance on the test set (data not shown). The one-hot encoded sequence, GSP and structural feature branches were built with 20/10 filters and 16/12 kernel sizes in the respective convolutional layers. The evolutionary conservation branch includes 32 filters for the first convolution layer and 16 for the second with kernel sizes 16 and 8 respectively. The range of values for the number of filters in both layers was 20, 30 and 40. The range for the kernel length was 20/15, 16/12 and 12/8. The fully connected part consists of 3 layers with 120/60/25 nodes, while the range of tested values was 140/80/40, 120/60/25 and 100/60/20. We selected leaky ReLU as the activation function of all layers followed by batch normalization. For the final layer that produces the algorithm’s output, we selected the sigmoid function.

All models were trained with binary cross entropy, for 60 epochs with the early stopper parameter enabled and a patience of 10 epochs. Hyperparameters were tuned by training different models for over 60 epochs exhausting all possible combinations, concluding in 0.001 learning rate with ‘Adam’ optimizer, batch size fixed at 256 and drop out rate of 0.2 (Additional file 1: Table S5). We tested models with 0.01, 0.001, 0.0005 and 0.0001 learning rates, 64, 128, 256 and 512 batch sizes and 0.2, 0.25, 0.3, 0.35 dropout rates. For the hyper-parameters tuning approach, a grid search has been utilized by learning for each of the hyper-parameter configurations and selects the best performing combination, over all.

### Feature extraction

Several GSP-inspired features were specifically selected as approximations of DNA’s physicochemical properties in the form of distinct time series. Z-Curve [39] comprises three signal vectors, each providing a unique representation of a DNA sequence. The three components x_n_, y_n_ and z_n_ correspond to an irrespective nucleotide distribution where x_n_ describes the distribution of purines/pyrimidines, y_n_ the functional group of the bases (amino or keto) and z_n_ the strength of the hydrogen bonds between base pairs (strong H-bond or weak H-bond). DNA-walk [40] describes a graph where a step upwards is taken if the current nucleotide is a pyrimidine and vice versa for a purine (does not take into account the previous nucleotide in contrast to Z-Curve). Paired numeric [41] incorporates the complementarity DNA sequences. The tetrahedron representation [42] is a fixed mapping method where all four nucleotides are considered to be the four vertices of a regular tetrahedron. DeepTSS also utilizes structural DNA features associated with promoter regions (Bendability and Propeller Twist) [43].

All spatial signals are calculated based on a 600 bp window around the CAGE peak representative. The window size has been decided based on the performance of multiple models trained with different values (data not shown), and its application was achieved with a stride of 1 bp. Z-Curve and tetrahedron representations are multidimensional signals composed of three vectors while DNA walk and paired numeric are one-dimensional vectors. These signal features are able to describe a genomic sequence, identify hidden periodicities and nucleotide distributions that can not be revealed with conventional methods.

Structural features were calculated by using a sliding window and converting each 600 bp sequence to overlapping 3-mers or 2-mers. For bendability, the input sequences were fragmented into overlapping 3-mers (1 bp stride), and for each 3-mer we assigned a score that was derived from earlier biochemical studies [44]. The dimensionality of the resulting vector was 598. The same strategy was applied for propeller twist [45, 46], with the only difference being that this feature is based on 2-mers. Therefore, the resulting vector had 599 values.

These features effectively transform the input DNA sequences into time series. In addition, each input sequence is also transformed into its one-hot encoded version and the phyloP-derived numerical representation of its evolutionary conservation. Regardless of the feature type, the application of the neural network convolutional process ensures that local patterns and more abstract combinations of them across different feature types that maximize the DL model’s capacity for distinguishing between positive and negative CAGE peaks will be identified.

### DeepTSS training

CAGE peaks and their representatives (N = 38,439) were extracted from the H9 sample, as described in the Overview of DeepTSS section, and used for training our model (Fig. 2b). Peaks that overlapped H3K4me3 and Polymerase II ChIP-Seq enriched loci and were positioned within 1 kb from annotated protein-coding TSSs, comprised the positive set (N = 11,304). Intergenic CAGE peaks that did not overlap with either H3K4me3 or Polymerase II bound regions formed the negative set (N = 11,579). Promoter-proximal CAGE peaks that were localized in regions flanking annotated promoters (9 kb in each direction and outside of the previously mentioned 1 kb window) were removed entirely from all analyses, to avoid the putative inclusion of functionally rich information in the negative set. We also generated a benchmarking set of 75,127 CAGE peaks (32,310 positives and 42,817 negatives) from the K562 sample which was used to query the generalization capacity of DeepTSS on data from a biological context that was not included in the training process, and to compare its performance with existing algorithms.

Chromosomes 15 and 14 were completely left out of the training process. The former was used for testing the models during the optimal hyperparameters search and the latter as an evaluation set during training. For all loci in the training and validation sets, a sliding window of 600 bp (the window based on which the input features are calculated) was used. Initially, the window was placed 100 bp upstream of the CAGE peak representative position and then moved to its final position 100 bp downstream, with a 25 bp stride. Therefore, for each sample in the initial set of CAGE peak representatives, we generated 7 additional samples where the input DNA sequence, based on which all input features are calculated, is not centered on the representative. With this approach, we augmented the input set by generating more samples and forced our model to remain unaffected by any potential biases regarding the position of the CAGE peak representatives within the input sequences [18].

### Application of existing algorithms

CAGER, RECLU, PARACLU and iTiSS were applied on the ctss files provided by FANTOM with default parameters and the results we considered as their positive predictions (Additional file 1: Table S4). Of the existing algorithms, TOMETOOLS and ADAPT-CAGE are the only methods that utilize ML to filter out noise from CAGE signal. ADAPT-CAGE and DiS-TSS were also used with default parameters (Additional file 1: Table S4). For TOMETOOLS, we downloaded 1,048,124 scored CAGE enriched loci, from all cell types profiled by FANTOM in the TSS_human from http://fantom.gsc.riken.jp/5/datafiles/phase1.3/extra/TSS_classifier. Liftover from UCSC was used to convert the coordinates to hg38. In this file, the information related to each CAGE peak cell-specificity is absent. To find TOMETOOLS predictions for the K562 cell line, we overlapped the TSS_human file with the K562 CAGE peaks (N = 47,377). This way, we generated TOMETOOLS predictions in H9 and K562 cells (Additional file 1: Table S1). The score threshold (0.228) was chosen based on the algorithm’s documentation and was used in the evaluation process shown in Fig. 3. In Fig. 5 benchmark, we applied multiple score thresholds.

It should be noted that due to the minor differences on the accepted input format and underlying processing mechanism of each algorithm, might lead to differences between the resulting CAGE output.

The computational times for ADAPT-CAGE, CAGER, RECLU, PARACLU and iTiSS are ~ 13 h, ~ 46 min, ~ 13 min, ~ 4 s and ~ 30 min respectively, for ~ 47,000 CAGE peaks. TOMTOOLS peaks were downloaded directly from the Fantom5 repository.

### Software requirements and benchmarking

DeepTSS was developed with Python 3.7, and TensorFlow (version 2.2) with Keras API (version 2.4.3) for implementing the DL part of the framework. All dependencies and a thorough documentation can be found at the GitHub repository.

To apply DeepTSS, users must provide a CAGE bam file or a bed file with precalculated CAGE peak representatives, the corresponding human genome assembly in fasta format, and the evolutionary conservation score as calculated by phyloP in bigWig format. If the phyloP score is not provided, the evolutionary conservation branch will not be used and predictions will be based on the other feature types.

DeepTSS was benchmarked in terms of computational cost on a computer running on an Intel Xeon E5-2630 v3 @ 2.40 GHz and a total of 8 threads was utilized for performance benchmarking, to simulate the average CPU capacity of personal computers. The time cost for predicting approximately 40,000 CAGE peak representatives was ~ 4 min on average, with all convolutional branches enabled.

## Supplementary Information

**Additional file 1: Tables S1–S6**. Evaluation results of all algorithms.

**Additional file 2: Figure S1**. Evaluation results of all algorithms for chromosome 15.

**Additional file 3: Figure S2**. Positive and negative zones around annotated genes.

## Data Availability

DeepTSS is implemented in Python and is freely available to the public on https://github.com/DianaLaboratory/DeepTSS.

## Abbreviations

CAGE: Cap analysis of gene expression
ChIP-Seq: Chromatin ImmunoPrecipitation followed by sequencing
CNN: Convolutional neural network
DL: Deep learning
GSP: Genomic signal processing
ML: Machine learning
NGS: Next generation sequencing
NN: Neural network
ReLU: Rectified Linear Unit
TFBS: Transcription factor binding site
TSS: Transcription start site
UCSC: University of California Santa Cruz
