# Supplementary figures and images for: DeepTSS: multi-branch convolutional neural network for transcription start site identification from CAGE data
Source: BMC Bioinformatics. 2022 Dec 12;23(Suppl 2):395. doi: 10.1186/s12859-022-04945-y (PMC9743497; doi:10.1186/s12859-022-04945-y)

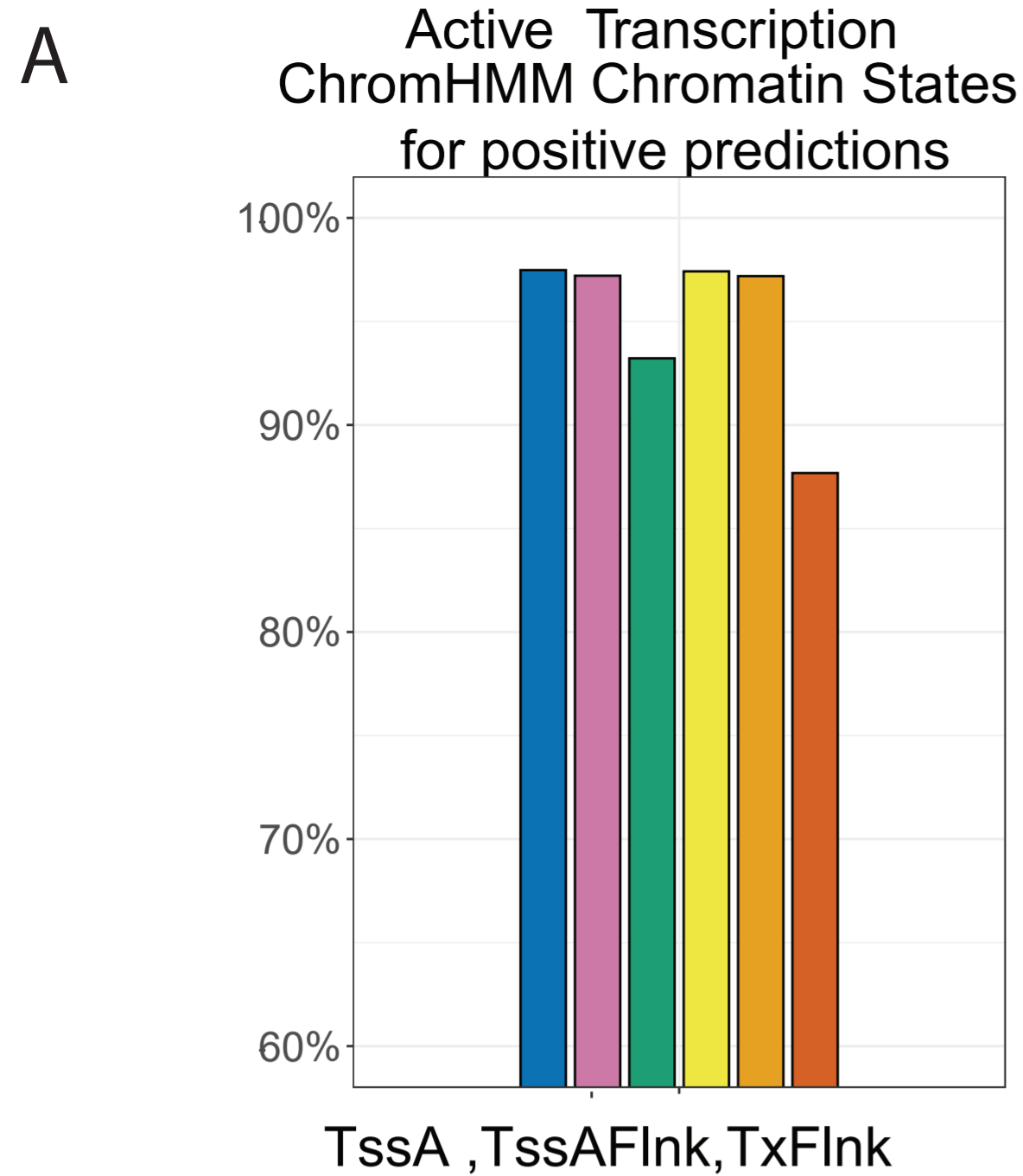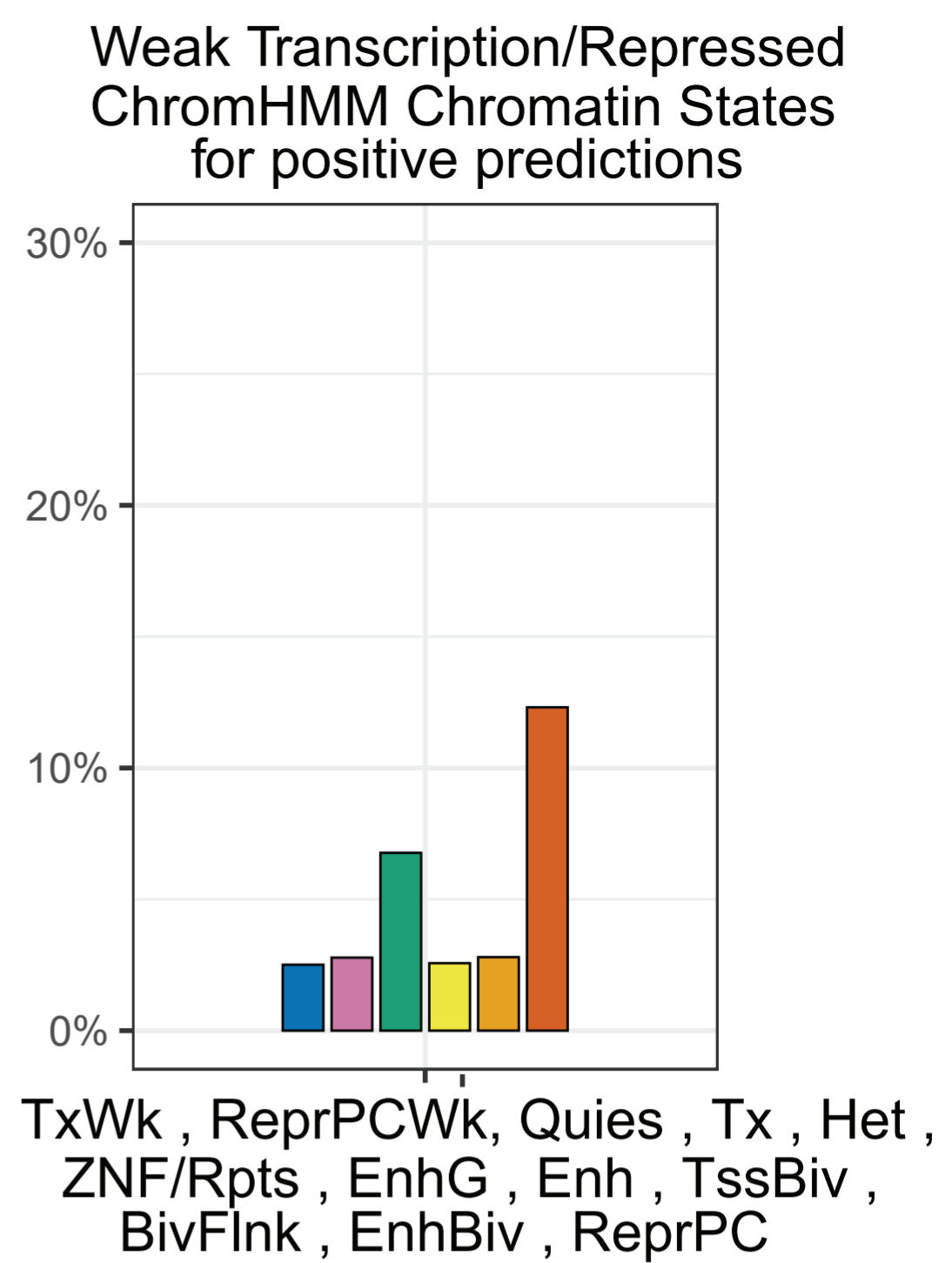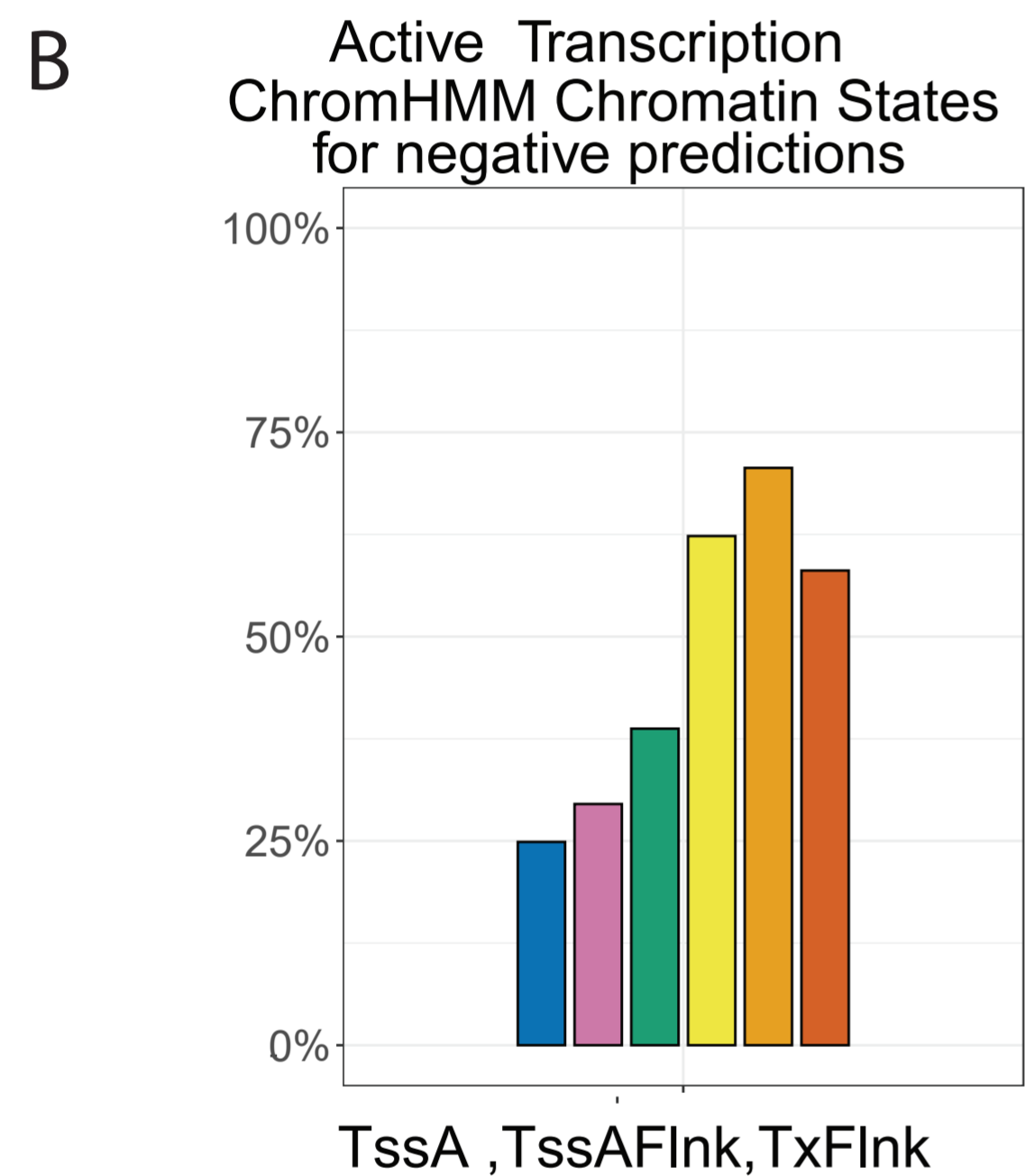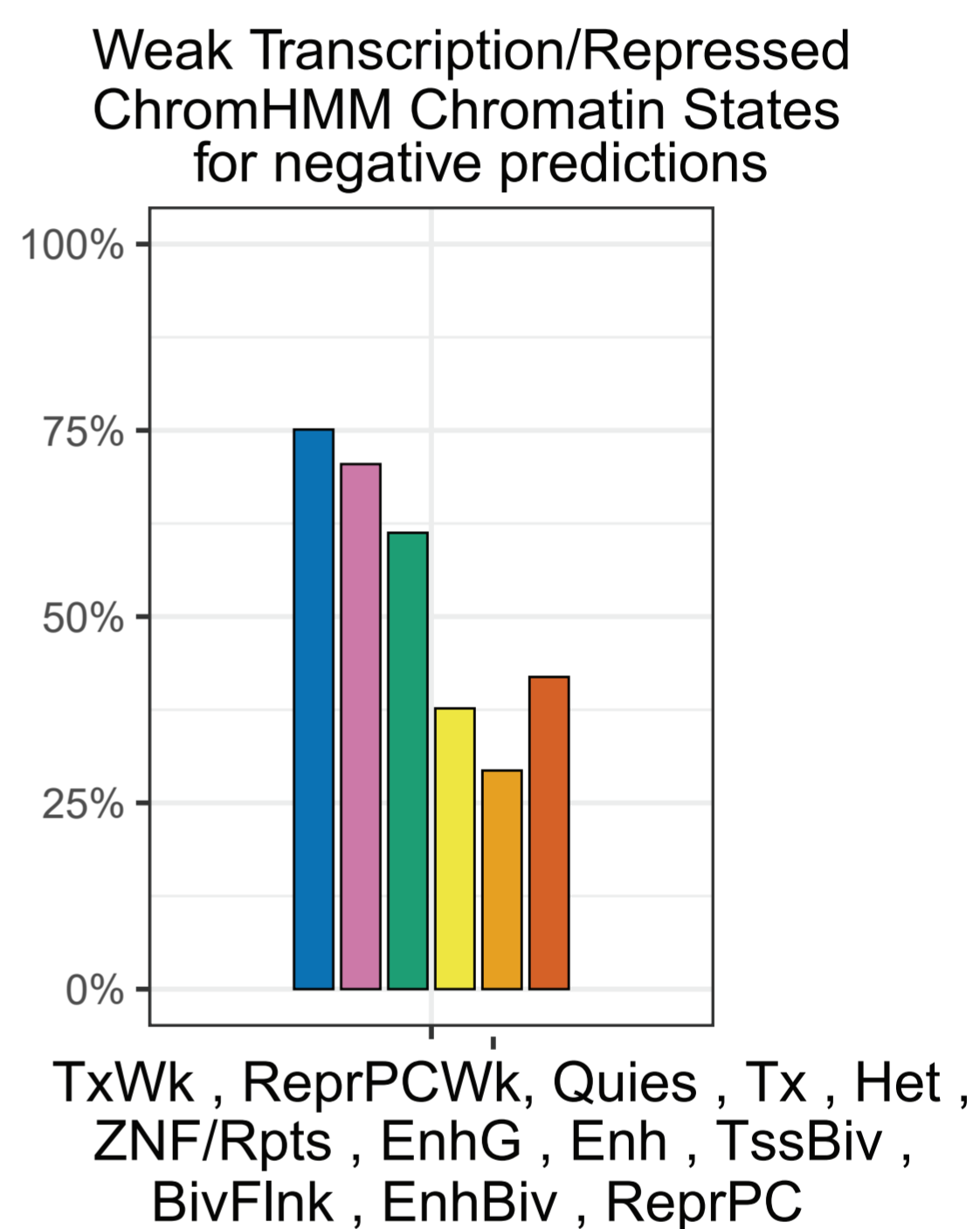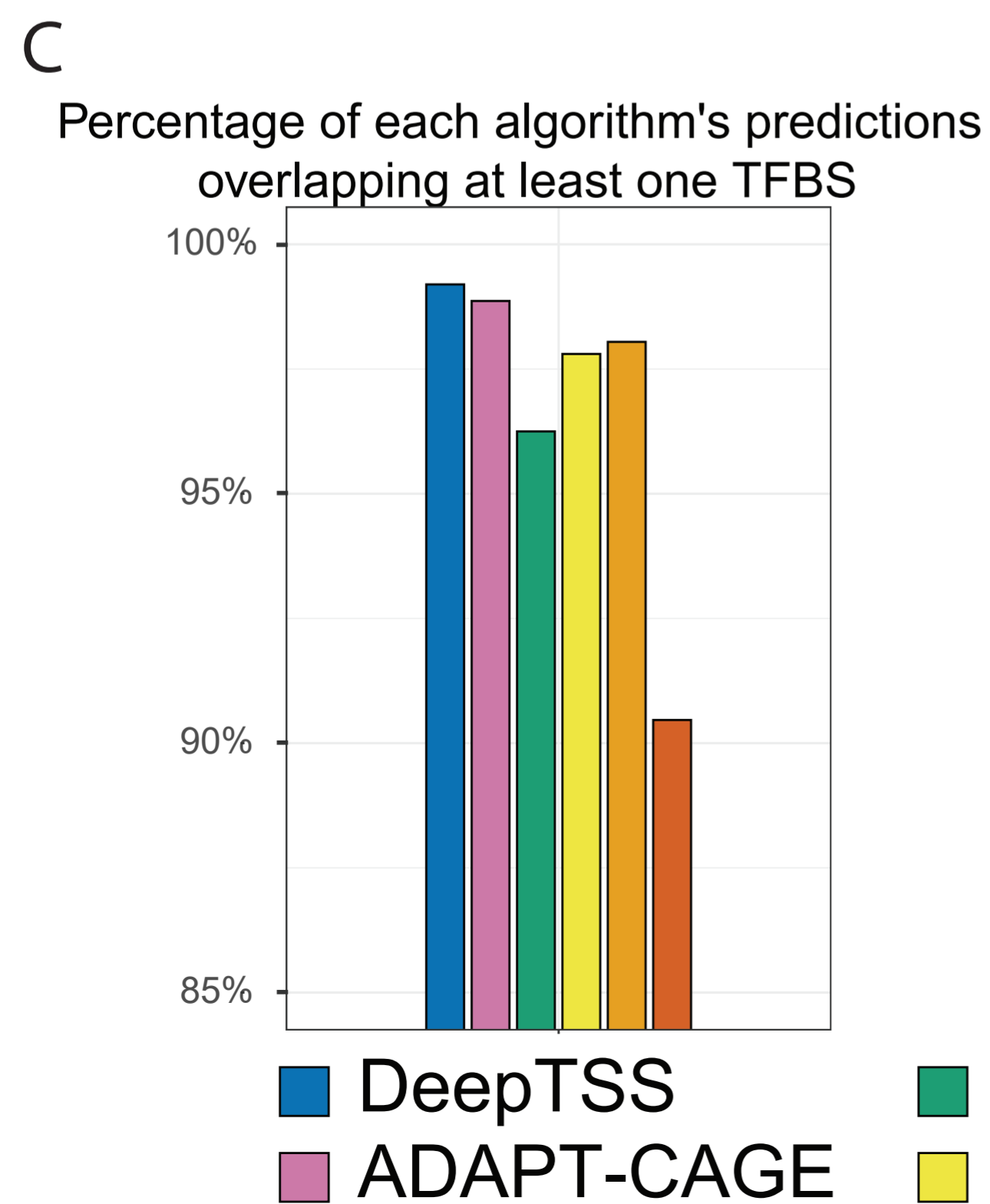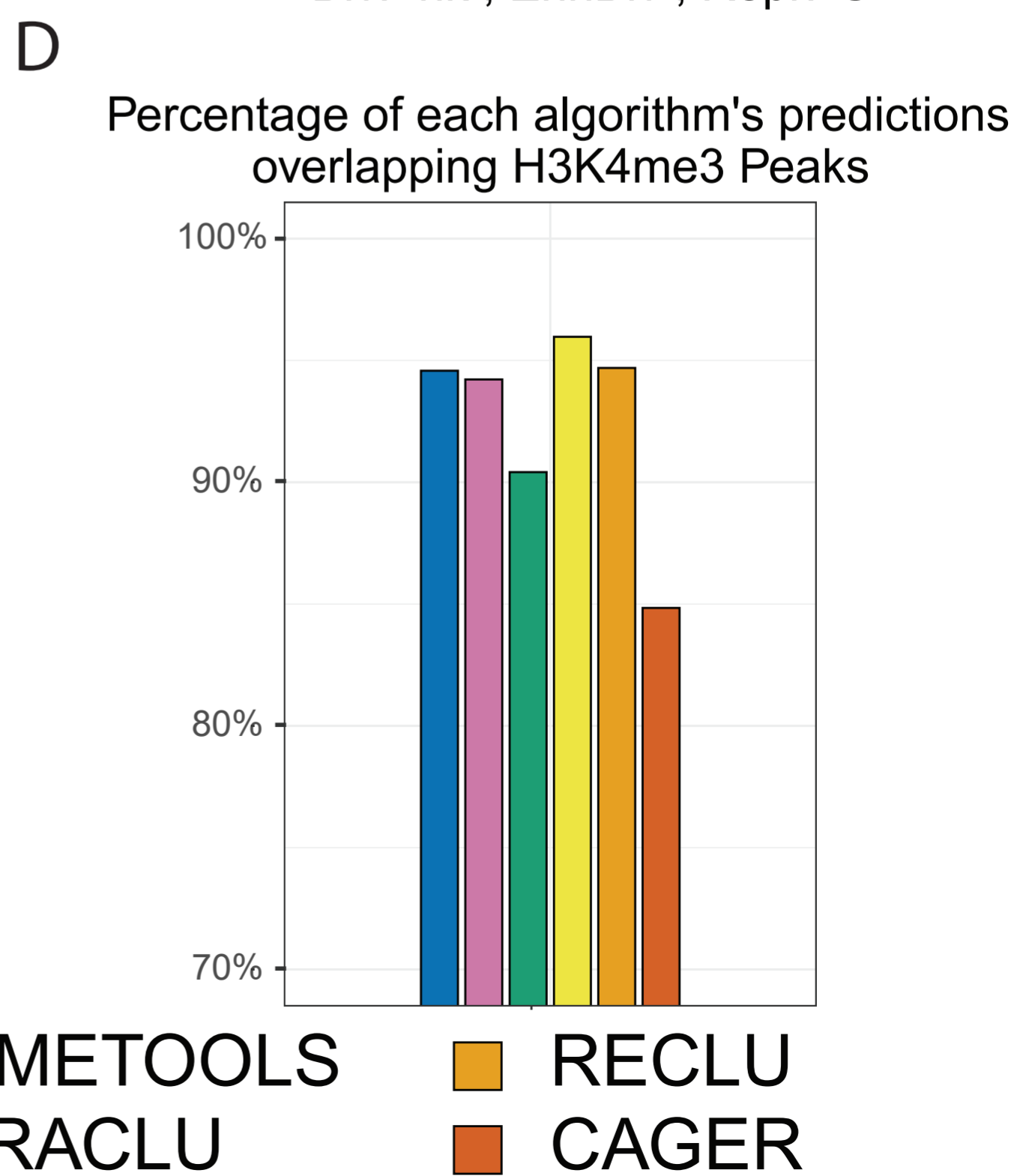

Supplement: Supplementary file 2 — Additional file 2: Figure S1. Evaluation results of all algorithms for chromosome 15. [file 12859_2022_4945_MOESM2_ESM.pdf]

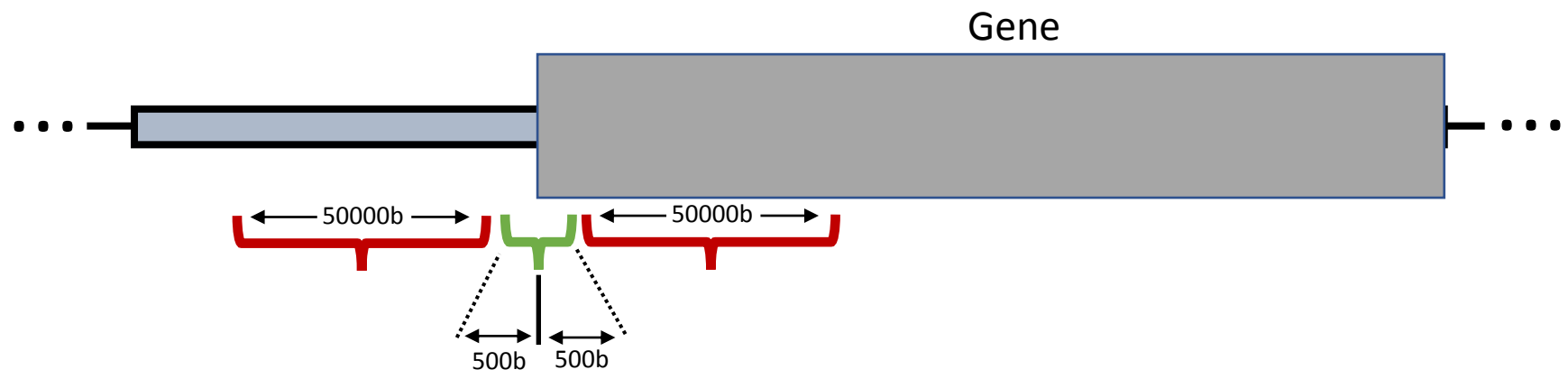

Supplement: Supplementary file 3 — Additional file 3: Figure S2. Positive and negative zones around annotated genes. [file 12859_2022_4945_MOESM3_ESM.pdf]
